# Phonology without universal grammar

**DOI:** 10.3389/fpsyg.2015.01229

**Published:** 2015-09-04

**Authors:** Diana Archangeli, Douglas Pulleyblank

**Affiliations:** ^1^Department of Linguistics, University of Hong KongPok Fu Lam, Hong Kong; ^2^Department of Linguistics, University of ArizonaTucson, AZ, USA; ^3^Department of Linguistics, University of British ColumbiaVancouver, BC, Canada

**Keywords:** linguistics, phonology, morphology of words, universal grammar, emergent properties, Esimbi, English, ultrasound and language

## Abstract

The question of identifying the properties of language that are specific human linguistic abilities, i.e., Universal Grammar, lies at the center of linguistic research. This paper argues for a largely Emergent Grammar in phonology, taking as the starting point that memory, categorization, attention to frequency, and the creation of symbolic systems are all nonlinguistic characteristics of the human mind. The articulation patterns of American English rhotics illustrate categorization and systems; the distribution of vowels in Bantu vowel harmony uses frequencies of particular sequences to argue against Universal Grammar and in favor of Emergent Grammar; prefix allomorphy in Esimbi illustrates the Emergent symbolic system integrating phonological and morphological generalizations. The Esimbi case has been treated as an example of phonological opacity in a Universal Grammar account; the Emergent analysis resolves the pattern without opacity concerns.

## 1. Introduction

In exploring the role of “Universal Grammar” in phonology, our starting point here is the observation in Deacon (1997) that “[l]anguages are under powerful selection pressure to fit children's likely guesses, because children are the vehicle by which a language gets reproduced.” (Deacon, 1997, p. 109). At issue is the source of those “likely guesses”: are they due to an innate capability specific for language, the *Universal Grammar* hypothesis (UG), or are they simply the abilities that infants use to learn about all aspects of their world, the *Emergent Grammar* hypothesis (EG)?

We know that humans perceive gradient information categorically, and that we are good at categorizing in general (e.g., Rosch et al., 1976; Zacks and Tversky, 2001; Zacks et al., 2006; Seger and Miller, 2010). We know that humans make use of Bayesian probabilities (e.g., Tenenbaum and Griffiths, 2001). And we know that infants are very aware of skewed frequencies in language (Maye et al., 2002; Gerken and Bollt, 2008; Dawson and Gerken, 2011). We know that humans create symbolic systems to represent their knowledge (Deacon, 1997). Under the Emergent Grammar hypothesis (e.g., Hopper, 1987, 1998; MacWhinney and O'Grady, 2015) the infant language learner is expected to make use of these abilities in understanding the language environment in which s/he is immersed.

a. Ability to create categories
b. Ability to attend to frequency
c. Ability to generalize and create a symbolic system

By stripping away aspects of phonological systems that can be determined by remembering items, categorizing them, attending to frequencies and creating symbolic systems, we will have a better understanding of the role of UG in phonology: UG is responsible for the residue that cannot be explained as emergent properties.

Consider the multiple tasks facing the infant learner on encountering language sounds. Among them are (a) the challenge of isolating specific sounds from the sound stream, (b) assigning specific sounds to sound classes, and (c) building a grammar to characterize the occurring sounds. Under both models, the first step involves grouping similar sounding sounds into single categories, a categorization task. After that point the two models differ. Under EG, the next step is a higher order categorization, identifying groups of sounds as similar in some way, such as articulation, acoustics, or behavior. (See Ellis et al., 2015 for a review of categorization and the internal structure of categories, particularly with respect to usage-based linguistic models. See Mielke, 2004 on why features cannot be innately defined, but must be learned.) This similarity leads to positing a category for that group of sounds: these categories correspond roughly to the familiar “distinctive features,” though there is no *a priori* set of features to map the sounds to, and in fact, a behavioral category is not necessarily an acoustic or articulatory category, and vice versa. The grammar involves further steps of abstraction, for example expressing observations about co-occurrences of feature categories (such as “all round vowels are back”).

In contrast, under UG, once sounds have been identified, a very different task arises, of mapping these sounds to an innate set of features. This is a challenge because the fit is imprecise: when we compare sounds across languages we see that there is a lot of variation in the realization of features (Lindau and Ladefoged, 1986; Ladefoged and Maddieson, 1996; Pulleyblank, 2006). Building the grammar is a further challenge: in addition to encoding observations about the behavior of the features that function distinctively in the language, the learner must also encode the directive to disregard features that do not function in the language. For example, under Optimality Theory, this would include learning which feature-constraints to promote and which to demote in the constraint hierarchy. The contrasts are summarized in Table 1.

**Table 1:** **The challenge**.

	task	Universal Grammar	Emergent Grammar

1.	“Sound” to sounds	Individuals to categories	Individuals to categories
			
2.	Sounds to types	Fit to pre-existing categories (i.e., features)	Categories are types
3.	Grammar	Rank constraints to prevent unfilled categories	(Further symbolizing)

In essence, under EG the learner's task is to work from concrete sounds to an increasingly symbolic system; under UG the tasks continually change, from categorizing, to mapping categories to abstract symbols, to organizing grammatical statements in a way that matches the observed categories (and creating those statements if they are not part of the genetic endowment).

In this paper, we explore the contribution of EG to the acquisition of adult grammars, looking first at the effects of categorization (English /ɹ/, Section 2), then examining the role of frequency (Bantu harmony, Section 3), and finally considering the impact of this approach on building a symbolic system, the overall morphophonological grammar (Esimbi prefixes, Section 4).

## 2. Categories and generalization

Mielke et al. (2010), Mielke et al. (accepted), Archangeli et al. (2011) report on a study of the articulation of American English /ɹ/, in different syllable-, consonant-, and vowel-contexts. It is well-known that there are both bunched and retroflex articulations of /ɹ/ in American English, where a retroflex /ɹ/ has the tip raised and the dorsum lowered and a bunched /ɹ/ has the tip lowered and the dorsum raised (Zhou et al., 2008); while some speakers use one articulation and some use the other, still others use both (Delattre and Freeman, 1968; Ong and Stone, 1998; Guenther et al., 1999; Campbell et al., 2010). Mielke et al. (accepted) demonstrates that for those speakers who use both bunched and retroflex articulations, the distribution of articulations is highly systematic for each speaker, and highly categorical.

Mielke et al. (accepted) observes that, by and large, subjects who use both bunched and retroflex do so categorically by environment. Interestingly, these environments are not shared across speakers. Rather, each speaker using both articulations has developed his/her own pattern of bunched and retroflex environments. Furthermore, there appears to be no evidence that the different articulations are perceptible, hence the speaker-specific systems appear to be “covert.”

A further point of interest is the nature of these covert grammars. The Table 2a shows Optimality Theoretic grammars (Prince and Smolensky, 1993; McCarthy and Prince, 1995; McCarthy, 2002) for four of the 11 such speakers. In each case, the constraint ^*^ɹ (“avoid bunched /ɹ/”) is ranked above ^*^ɹ (“avoid retroflex /ɹ/”) encoding the fact that these speakers preferred retroflex /ɹ/ to bunched /ɹ/ except in a specific set of environments. The several constraints that outrank “avoid bunched” provide the simplest characterization for each speaker of the special environments where /ɹ/ is bunched, not retroflex. As inspection of Table 2a reveals, there is a high degree of similarity in these grammars, but none are the same. (See Mielke et al., accepted for discussion of the phonetic properties of the conditions governing the two articulations of /ɹ/.)

**Table 2:** **Covert and overt systems: column headings in (a) indicate general properties of the relevant contexts; individuals implement the contexts in different ways**.

a.	Covert Am. Eng. ɹ grammars (Mielke et al., accepted)									
		codas	[i]	Coronals/[θ]	[ʃ]	[k]				

	i.	^*^coda _ɻ_	^*^_ɻ_ i	^*^θ_ɻ_ o	^*^ʃ_ɻ_ o	^*^k_ɻ_ a	>>	^*^ł	>>	^*^_ɻ_
	ii.	^*^coda _ɻ_	^*^_ɻ_ i	^*^θ_ɻ_		^*^k_ɻ_	>>	^*^ł	>>	^*^_ɻ_
	iii.		^*^_ɻ_ i, ^*^i_ɻ_	^*^_ɻ_[Cor]	^*^ʃ_ɻ_	^*^ɑ_ɻ_k	>>	^*^ł	>>	^*^_ɻ_
	iv.	^*^coda _ɻ_	^*^_ɻ_ i		^*^ʃ_ɻ_		>>	^*^ł	>>	^*^_ɻ_
b.	Overt systems: 4 /l/ allophony grammars (Mielke et al., accepted)									
		language		adjacency restrictions						

	i.	Boumaa Fijian		^*^lu			>>	^*^ł	>>	^*^l
	ii.	Buriat		^*^łʃ			>>	^*^l	>>	^*^ł
	iii.	Assyrian Neo-Aramaic		^*^C^ʕ^l, ^*^lC^ʕ^			>>	^*^ł	>>	^*^l
	iv.	Alabama		^*^ulC	>>	^*^lC	>>	^*^ł	>>	^*^l

In contrast, compare the grammars for dark and light /l/ in four languages where the distinction is allophonic (of 17 languages reported on), Table 2b. In these cases, the pattern is a characteristic of the language, not of individual speakers. Most striking is the relative simplicity of these four overt systems, with only one or two constraints outranking the core grammar defining how /l/ is articulated in each language.

### 2.1. Summary

While we see that the overt systems have simplified rules/constraint hierarchies, possibly to make them more robustly identifiable, of real interest here are the covert systems. The patterns of covert /ɹ/ allophony show the categorical use of distinct articulations. More importantly, we see that individuals make individual generalizations *even in the absence of consistent input in the environment*: Categorization and generalization into a symbolic system is simply what humans do when encountering data. (For further discussion, see Archangeli, 2009).

These observations are consistent with the conclusion that humans are driven to generalize. These generalizations are based on data available to the learner but may go beyond observable patterns. Shared patterns result when certain sound types or sound sequences have an observable skewed distribution, the topic of our next section.

## 3. Frequency and generalization

A key difference between UG and EG is that UG leads us to expect categorical effects: a rule applies, or it does not apply; a constraint is dominant, or it is subordinate. Under this categorical approach, arbitrary exceptions are troubling, yet it is well-known that language is “messy.” This has led to models which assume UG yet abandon strict categorical behavior by allowing exceptions to rules (e.g., Chomsky and Halle, 1968) and to constraint-based models which assign values to constraints without imposing discrete ranking (e.g., Legendre et al., 1990; Boersma, 1997; Boersma and Hayes, 2001; Goldrick and Daland, 2009; Pater, 2009). In contrast, exceptions are expected and normal under the Emergentist model because frequency does not require absolute, categorical behavior, but simply a skewed distribution in order to identify a pattern.

In this section, we present evidence showing that frequency data is consistent with EG, not with UG. The discussion is based on Archangeli et al. (2012b).

Archangeli et al. (2012b) considers three differing predictions of the UG and EG models. We address two of them here, summarized in Table 3. First, how well do the data match the rules/constraint rankings of the language? We call this “goodness of fit”; UG predicts very few exceptions to rules/constraint rankings, so the data should fit the grammar very tightly. EG, by contrast, builds a grammar from the bottom up, so predicts a range of fits from tight to loose even within the same grammar. Second, the bottom-up EG model means that the learner is figuring out phonological patterns—and making classificatory errors—even before morphological categories are established. Consequently, EG predicts that a pattern that is morphologically restricted to one domain will gradiently extend into other domains (e.g., a pattern restricted to verbs will nonetheless be found, though to a lesser extent, in nouns). UG predicts the absence of extension into other morphological domains: the rules/constraints are defined and exceptions should not occur. (Similarly a pattern that is phonologically restricted is expected under EG to extend to a broader phonological domain, a prediction UG does not make. We suppress that discussion here in the interests of space; see Archangeli et al., 2012b for details.)

**Table 3:** **Predictions: UG vs. Emergence (Archangeli et al., 2012b)**.

	UG	EG

Goodness of fit	tight	loose
Gradient extension of morphosyntactic domain	no	yes

This study required data with very specific properties. In addition to identifying languages with some pattern having both morphological and phonological restrictions, the languages had to be organized into searchable databases and there needed to be comparable control languages.

An appropriate pattern was found with Bantu height harmony. In many Bantu languages, verb suffixes alternate between high vowels and mid vowels, with the mid vowels occurring after other mid vowels. The pattern is described as morphologically restricted to verbs. It is also phonologically asymmetric, with [e] typically not followed by a high front vowel (^*^e…i) and with [o] not followed by both front and back high vowels (^*^o…i, ^*^o…u). The paradigm in Table 4 illustrates the pattern.

**Table 4:** **Bantu Height Harmony in Ciyao (Ngunga, 2000)**.

	‘-il’ applicative			‘-u’ reversive		
	
a.	dim-	dim-il-	*cultivate*	siv-	siw-ul-	*close/open up*
b.	wut-	wut-il-	*pull*	uuv-	uuw-ul-	*hide/reveal*
c.	saam-	saam-il-	*move*	mat-	mat-ul-	*adhere/peel off*
				
d.	pet-	pet-el-	*ornament*	sweek-	sweek-ul-	*insert/pull out*
				
e.	soom-	soom-el-	*read/study*	som-	som-ol-	*pierce/extract*

The harmonic pattern leads to an expected skewing of the distribution of vowels in these languages: we expect even distribution of all V_i_… V_j_ sequences except with three sequences, *e…i, o…i*, and *o…u*. Each of these three sequences is unexpected in test-case verbs but expected in the other two environments, Bantu nouns and the control languages. Archangeli et al. (2012b) focuses specifically on these three sequences.

Relevant data sets of Bantu languages with a five vowel system [i, e, a, o, u] and height harmony are found in Bukusu, Chichewa, Ciyao, Ikalanga, Jita, and Nkore-Kiga, in the Comparative Bantu OnLine Dictionary (CBOLD: http://www.cbold.ish-lyon.cnrs.fr/). Control cases (with the same five vowels and no harmony) were found in *freelang.net* (http://www.freelang.net/): Ainu, Fulfulde, Hebrew, Japanese, Kiribati, and Maori.

In the test words, Archangeli et al. (2012b) counted sequences of two vowels, V_1_…V_2_ for all V_1_, V_2_, ignoring intervening consonants in all words and ignoring prefix vowels in the test languages (because the harmonic pattern does not extend to prefixes). These counts were used to determine the expected distribution of V_1_…V_2_ sequences for each V_1_…V_2_ pair in each language; for test languages, the data were further subdivided into nouns and verbs. Comparing the observed with the expected distributions (*chi* square, with observed/expected ratios converted to log_2_ values) revealed which sequences were over-represented and which were under-represented. As noted above, of special interest are the sequences *e…i, o…i*, and *o…u*, each of which is expected to be underrepresented, given the harmony pattern. A value of 0 shows distribution as expected; negative values show under-represented sequences (−1 appears half as often as expected, etc.) and positive values show over-representation.

In determining goodness-of-fit, a tight fit for a disallowed pattern is shown by extremely negative values (non-occurrence is -∞), while a loose fit is shown by somewhat negative values. In all three cases, the control language averages are very close to 0, while the verbs in test languages average significantly below 0.

At the same time, each of these key sequences *is* found in verbs, in some if not all of the test languages. As Archangeli et al. (2012b) shows, there are only three languages where one of the sequences is not found in the verb sample. In all three cases, the unattested sequence is *o…u*; it is not found in Chichewa, Ciyao, or Nkore-Kiga. A sequence like *e…i*, in contrast, is rare but does occur occasionally; for example, Ciyao has verb stems like *-nyésíma* “glitter” and *-gwésima* “be dullwitted,” exceptions to the general prohibition against a mid vowel followed by a high front vowel.

A close, tight fit between data and generalization would show no occurrences of these sequences in any of the languages. But in all cases, while the distribution of the key sequences in verbs is well-below the 0-line, the distance from the 0-line varies by language and by vowel sequence. In short, we do not see the tight fit predicted by UG; instead we see gradient adherence to the pattern as predicted by EG.

The expectation with morphological extension under EG is that the distribution of the three key sequences will also be depressed in nouns (less than 0, but greater than the verbs); UG expects these sequences to show normal random distribution (near 0). The facts support the EG hypothesis: There is a skewing toward under-representation of these sequences in nouns, though it is not as pronounced as in verbs. Furthermore, the more skewed the verb sequence is, the more skewed the noun sequence as well.

In this section, we have summarized the argument in Archangeli et al. (2012b), that frequencies of V…V sequences in the Bantu show a loose fit to the pattern, and a gradient extension of the morphosyntactic domain, precisely as predicted in Table 3 by a minimal innate linguistic endowment for phonology, the Emergentist model.

Archangeli et al. (2012a) goes a step further, expressing prohibited and preferred sequences as conditions; over-represented sequences such as *e…e* and *o…e* lead to the promotion of conditions such as *if V_1_* = [e, o] then V_2_ = [e], while under-represented sequences such as *e…i* and *o…i* do not induce promotion of some condition, etc. These conditions express the grammatical generalizations that phonologists converge on, and so provide a means of discovering phonological patterns in a language without appeal to innate constraints or constraint (or rule) schema. From these demonstrations, we conclude that the language-learning infant can discover and express phonological patterns in their language without appeal to innate linguistic universals, at least in the kinds of cases considered: The general strategy of attending to the frequency of different sequences leads to identifying and symbolizing patterns.

Our goal to this point has been to demonstrate the merit of Emergent Grammar: the predictions of EG fit the data better than do the predictions of UG. We turn now to a very different type of question, namely, the implications of EG for other aspects of grammar. That is, does the nature of an analysis change significantly if we adopt EG? In the next section, we argue that there are clear differences in the way a language is represented.

## 4. Implications for grammars

In this section, we explore the prefix vowel patterns in Esimbi, a Tivoid language, a member of the Bantoid branch of Niger-Congo (Stallcup, 1980a,b; Hyman, 1988; Coleman et al., unpublished manuscript; Kalinowski, unpublished manuscript; Koenig et al., unpublished manuscript; Stallcup, unpublished manuscript).^1^ While the surface vowels of roots do not alternate, some prefix vowels do alternate, depending largely on the root to which they are attached. Esimbi vowels are given in Table 5a, and the forms in Table 5b show no surface trigger for the difference in prefix vowel height: the class 7, 8, 9, and 10 prefixes are high with the roots for “bone” and “back” but mid with the roots for “belly” and “cane rat.”^2^

**Table 5:** **Interactions with morphology in Esimbi**.

a.	*Vowels*			b.	*Opaque prefix selection*		
	i	ɨ	u		Sg7	Pl8	
					
	e		o		[ki-ku]	[mi-ku]	‘bone’
	ε		ɔ		[ke-tɨmbɨ]	[me-tɨmbɨ]	‘belly’
		a					
					Sg9	Pl10	
					
					[ì-jìmì]	[í-jimi]	‘back’
					[è-b1]	[é-b1]	‘cane rat’

A standard generative approach to the pattern would be to assign underlying height values to roots, cause prefixes to harmonize with roots in terms of height, and then to neutralize all root vowels to high (see, e.g., Hyman, 1988). This results in surface opacity under the assumption that the prefix height is a phonological alternation because there is no surface phonological trigger for the prefix alternation. Under EG, we ask first what the learner is likely to generalize based on frequency of category distributions. We then turn to the question of whether these generalizations resolve the opacity problem.

### 4.1. Root properties

Without going into detail here, we assume that identifying morphs and classes of morphs in a concatenating language like Esimbi is a challenge that the learner has faced and overcome. (See Archangeli and Pulleyblank, 2012, Forthcoming 2016a,b for those details.) We start here with the point at which the learner has already started identifying nouns and verbs as distinct from each other, and is noting that phonologically different forms of verbs appear with different meanings. This enables the learner to identify, for a sequence such as *uri*, that there is a verb root, *ri* “eat,” and an infinitival marker *u*.

As the data in Table 6 show, verb roots vary in length from 1 to 3 syllables. However, despite the 8 vowels in the vowel inventory, Table 5a, verb roots are restricted to a limited set of vowels, the high vowels [i, ɨ, u]. Furthermore, the vowels in a verb root are overwhelmingly identical, all [i], all [ɨ], or all [u], but no combinations. In short, root vowels are high; root vowels agree in frontness and in rounding. This pattern is further confirmed by inspection of nouns, representative examples given in Table 7, which shows that this distribution of height and identity holds of all roots, not just of verbs.

**Table 6:** **Verbs with infinitive prefix (Hyman, 1988) (tone not included in source)**.

	[i] roots		[u] roots		[ɨ] roots	

	u-ri	‘eat’	u-zu	‘kill’		
u	u-bini	‘dance’	u-tumu	‘send’		
	u-fihiri	‘dangle’	u-suhuru	‘crouch’		

	o-si	‘laugh’	o-tu	‘insult’	o-dzɨ	‘steal’
o	o-kibi	‘pour’	o-zumu	‘dry up’	o-tɨnɨ	‘refuse’
	o-yihiri	‘learn’	o-yuwuru	‘hear’	o-nɨmɨnɨ	‘think’

	ɔ-ri	‘daub’	ɔ-hu	‘knead’	ɔ-bɨ	‘come’
ɔ	ɔ-rini	‘be poor’	ɔ-buru	‘be tired’	ɔ-nɨmɨ	‘bite’
	ɔ- 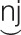 ihiri	‘chew’	ɔ-zumulu	‘wither’	ɔ-sɨ 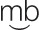 ɨrɨ	‘scatter’

**Table 7:**
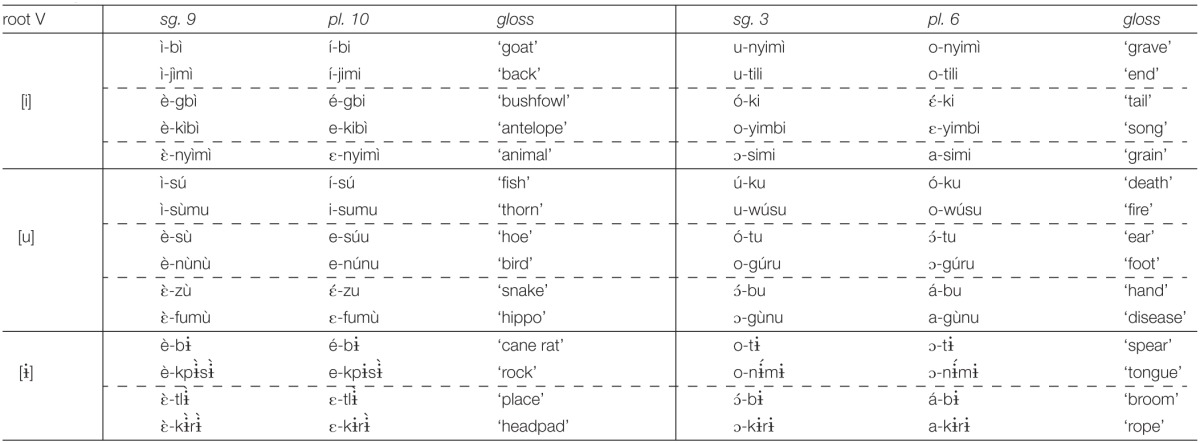
**Noun prefix and root vowels (Hyman, 1988, pp. 258–259)**.

[Typos in the tones of ‘fish’ and ‘hoe’ in Hyman (1988) have been corrected (Larry Hyman, p.c.).]

Review of prefixes in Esimbi shows that any of the eight vowels may occur as a prefix, one property that distinguishes them from roots. (The vowel [ɨ] occurs only in invariable prefixes, not in the prefixes that alternate; our focus is on the alternating prefixes.) Our first set of generalizations, a–e below, captures the restrictions on roots, restrictions that do not extend to prefixes. We express the sequential conditions as unbounded restrictions on particular feature sequences (Smolensky, 1993; Pulleyblank, 2002; Heinz, 2010). We also assume that generalizations about the sounds of the language include statements like ^*^[front, round], etc.; we do not include these statements in our discussion.

a. ^*^[nonhigh]_root_
b. ^*^[back]…[front]_root_
c. ^*^[front]…[back]_root_
d. ^*^[round]…[nonround]_root_
e. ^*^[nonround]…[round]_root_

### 4.2. Prefix distribution

The prefixes are far more challenging. In Tables 6, 7, we see that the correct form of the prefix depends in part on the particular prefix (e.g., the infinitive prefix is back and rounded, one of [ u, o, ɔ ], while the singular 9 prefix is front and unrounded, one of [ i, e, ε ], etc.), while selection of a specific morph from within each prefix set depends on which root the prefix is attached to.

In figuring out the morphs of Esimbi, a further set of generalizations is possible, relating prefix morphs to each other. This set of generalizations is definitive in some cases, shown in *f–i*, but in other cases options are available, as in *j–l*^3^.

f. If a prefix has a morph { i }, it also has morphs { e, ε }.
g. If a prefix has a morph { e }, it also has morphs { i, ε }.
h. If a prefix has a morph { u }, it also has morphs { o, ɔ }.
i. If a prefix has a morph { a }, it also has morphs { o, ε ɔ}.
j. If a prefix has a morph { ε }, it also has morphs { i, e } *or* { o, ɔ, a }.
k. If a prefix has a morph { o }, it also has morphs { u, ɔ } *or* { ε, ɔ, a }.
l. If a prefix has a morph { ɔ }, it also has morphs { u, o } *or* { o, ε, a }.

Lexical generalizations of this sort are potentially useful to the learner: when a new form is encountered, it is possible to “fill in the blanks” in the lexicon. Thus, if a new form with an [i] prefix is encountered, the learner anticipates items with [e] and with [ε] as the corresponding prefix.

Which prefix morph is selected depends on the root to which the prefix is attached, as summarized in Table 8. An examination of these patterns establishes that roots need to be partitioned into three sets, A, B, and C, corresponding to the three rows in Table 6 and to the partitions within the three “root V” blocks in Table 7. As summarized in Table 8, a Set A root selects the highest/most advanced morph possible: {*i*, e, ε }; { *u*, o, ɔ }; { *o*, ε, ɔ, a }; Set C roots select the lowest/most retracted morph possible: { i, e, ε }; { u, o, ɔ}; { o, ε, ɔ, *a*}. Set B selects morphs that are not peripheral in terms of height among the possible morphs: { i, *e*, ε }; { u, *o*, ɔ}; { o, ε, ɔ, a}.

**Table 8:**
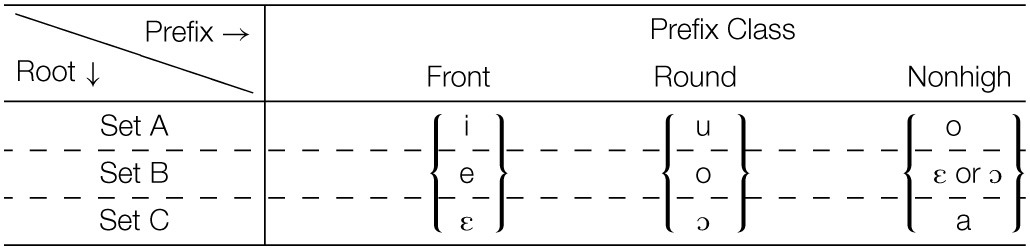
**Esimbi prefix descriptive summary**.

As this point, we have identified lexical properties of both prefixes and roots in Esimbi. Roots are assigned to one of three sets, A, B, C, and as far as we can tell, the assignments are arbitrary. That is, there is no phonological property of a root that could be used to determine which prefix occurs with that root. Prefixes are identified as a collection of morphs. What remains is to identify the generalizations by which roots select the appropriate morph from each set^4^.

### 4.3. Esimbi prefix selection

The general strategy we propose when selecting among alternatives is to identify the form that best fits whatever requirements there are for a given situation; for Esimbi prefixes, that means selection of the morph that best fits the requirements of the root to which it is attached. Essentially, with Set A roots, the root prefers a high and advanced vowel if possible, while with Set C roots, the preference is for a retracted vowel, preferably low. With Set B roots, the root gives no guidance and so the most representative morph of the set is selected.

#### 4.3.1. Set A roots: Prefer high advanced vowels

Consider first roots of Set A, exemplified in Table 9i (there are no Set A roots with the root vowel [ɨ]). Set A roots require the highest, most advanced morph of the set. The key generalization for Set A roots is that these roots prefer that a prefix be high and be advanced, Table 9ii. As laid out in Archangeli and Pulleyblank (2015), the grammatical expression of this kind of preference is part of the lexical representation of the verb roots. For Esimbi, Set A is defined by a specified preference for a preceding high vowel and a preceding advanced vowel, Table 9iii.

**Table 9:**
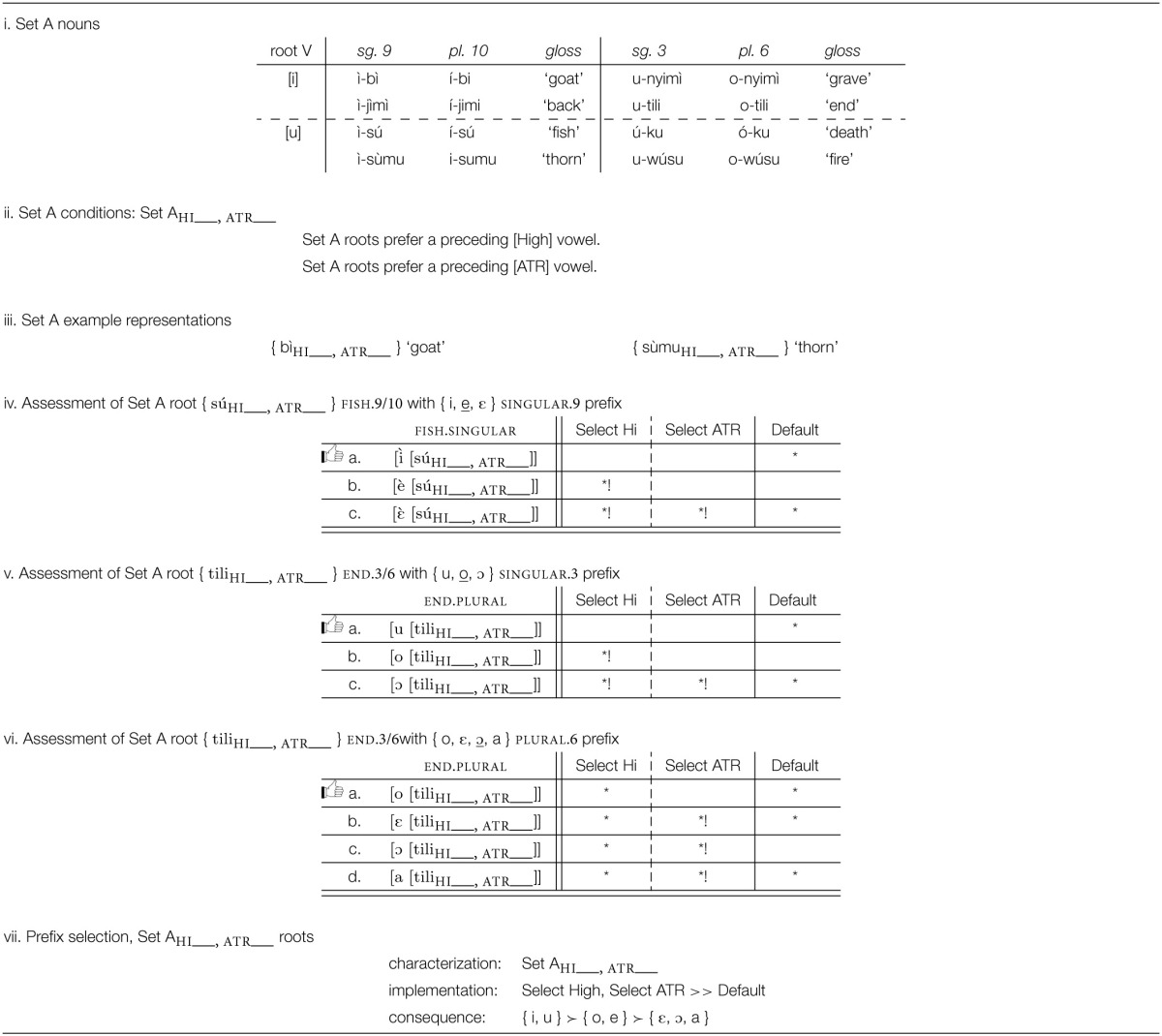
**Analysis of prefix selection for Esimbi Set A words**.

Where the prefix morph set includes a vowel that is high and advanced, that vowel is selected because it is a perfect match: as shown in Table 9iv, the prefix} { i } is selected over other members of the morph set { i, e, ε }, and as shown in Table 9v, { u } is selected out of { u, o, ɔ }. If the prefix morph set does not contain a high advanced vowel, as with the morph set { o, ε, ɔ, a }, then an advanced vowel is the best selection possible, as shown in Table 9vi. Defaults (underlined) are discussed in Sections 4.3.3, 4.3.4.

Our formal representation of selection, shown in Table 9 as well as in Tables 10–12, bears similarities to Optimality Theoretic tableaux (Prince and Smolensky, 1993; McCarthy, 2002). Differences lie in the nature of constraints (learned vs. innate) and the “candidate set” (the Cartesian product of relevant morph sets vs. an infinite set). Tables like those in Tables 9iv–vi are interpreted in a fashion similar to Optimality Theory tableaux (Prince and Smolensky, 1993), with the following differences. First, the upper left cell shows the morpho-syntactic features to be manifested in a phonological form (see Archangeli and Pulleyblank, Forthcoming 2016a for more on this point). The conditions across the top row are the conditions learned based on exposure to data; they are not innate “universals.” The possibilities in the lefthand column are all logically possible combinations of the relevant morphs—a finite set limited by the Cartesian product of the morphs involved (not an infinite set as in Optimality Theory). As with Optimality Theoretic tableaux, dashed vertical lines show unranked conditions and solid vertical lines show critical rankings; the symbol ^*^ is used to show when a form does not satisfy a particular condition and ^*^! shows crucial violations that eliminate a form from consideration. The thumbs up (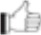) indicates the form selected, given the morphs and conditions. See (Archangeli and Pulleyblank, Forthcoming 2016b) for deeper comparison and contrast.

**Table 10:**
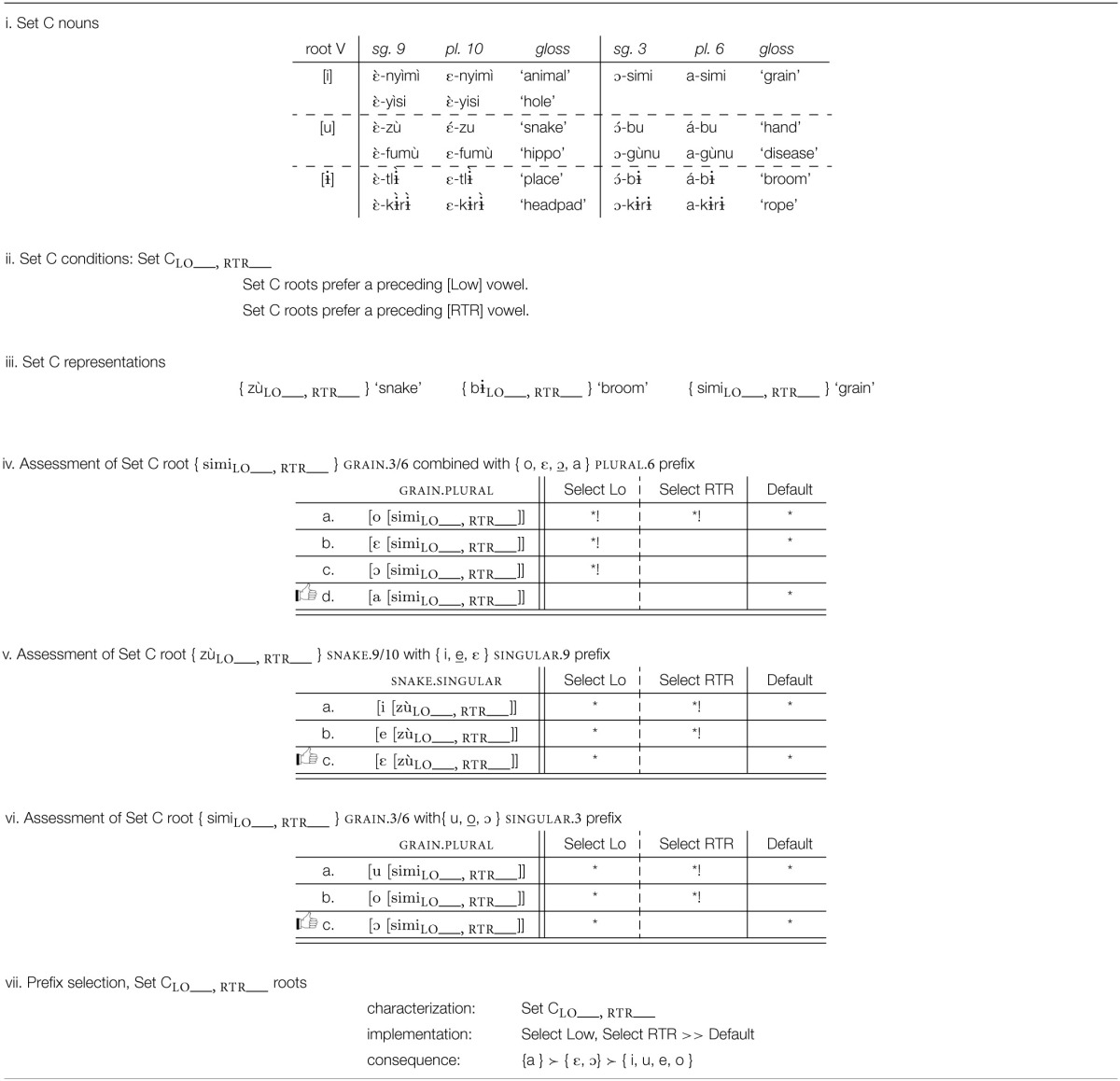
**Analysis of prefix selection for Esimbi Set C words**.

**Table 11:**
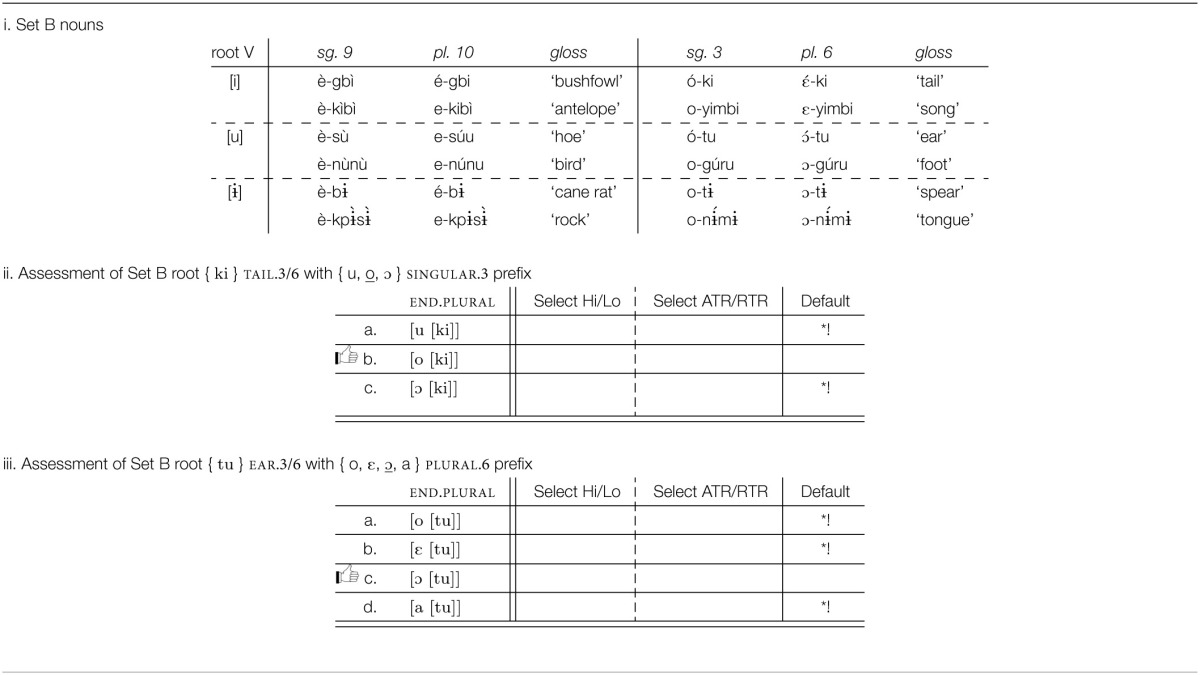
**Analysis of non-low prefix selection for Esimbi Set B words**.

**Table 12:**
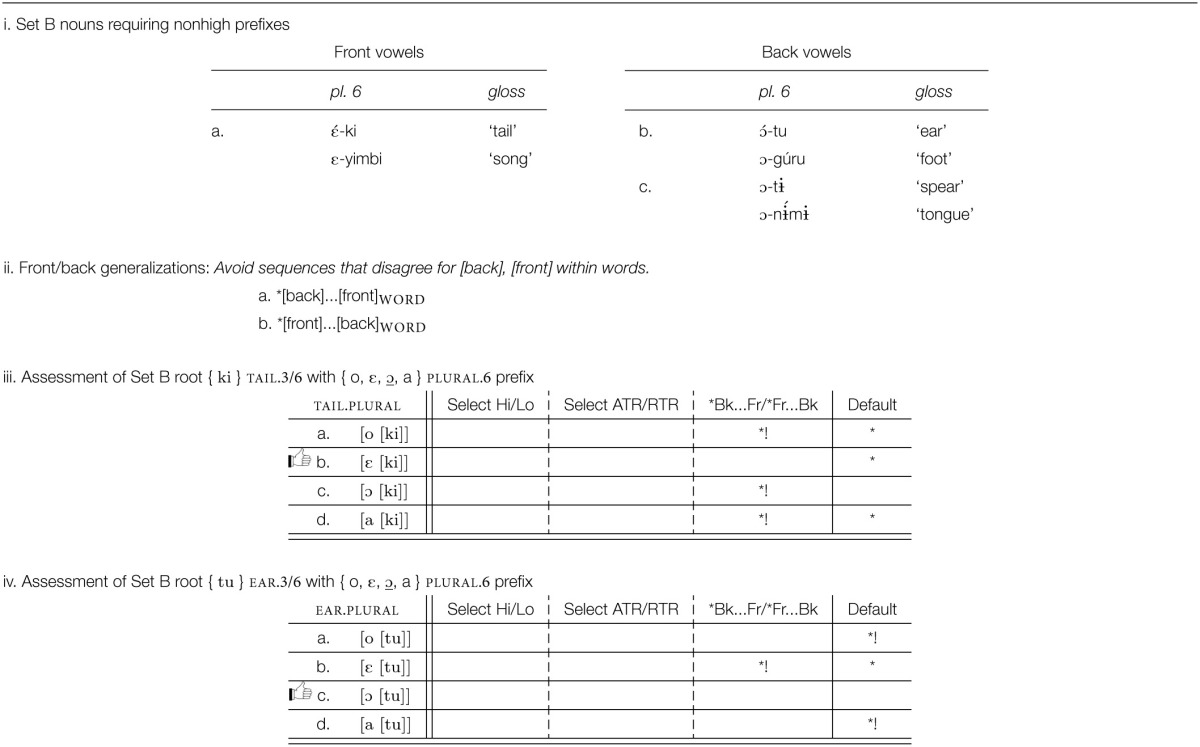
**Analysis of prefix selection for Esimbi Set B words and the prefix set with the low vowel option**.

The selection generalization and the implementation of best-fit are summarized in Table 9vii.

#### 4.3.2. Set C roots: Prefer low retracted vowels

With Set C roots, the analysis is very similar; the key difference is that these roots select for low, retracted vowels in their prefixes. Examples are given in Table 10i. In this case, the generalization is that low retracted vowels are preferred. In the absence of a low retracted vowel, either low or retracted vowels are preferred. This selects { a } over { o, ε, ɔ }, { ε } over { i, e }, and { ɔ } over { u, o }. Set C is defined and exemplified in Tables 10ii–iii.

Where the prefix morph set includes a vowel that is low and retracted, that vowel is selected because it is a perfect match: { a } is selected over { o, ε, ɔ }, as shown in Table 10iv. If the prefix morph set does not contain such a vowel, as with the morph sets { i, e, ε } and { u, o, ɔ }, then a retracted vowel is preferred to the two advanced vowels, as shown in Tables 10v,vi, respectively. The selection generalization and the implementation are summarized in Table 10vii.

The preference for low retracted vowels selects { a } for Class 6, the one prefix morph set with a low retracted vowel. In the other two prefix morph sets, there is no low-voweled morph, and the next best thing is a match for the retracted feature, selecting { ε } for Class 9–10 and { ɔ } for Class 3.

#### 4.3.3. Set B roots: Phonological, but not morphological, selection

Selection of the prefix morph for B roots is a bit more interesting, involving both selection of a “default” morph and an interaction of phonological sequencing restrictions with morph selection. We consider the default effect first. Set B nouns are illustrated in Table 11i.

We propose that Set B roots place no restrictions on morph vowels, leaving the selection to be determined for each affix by other criteria, such as the properties of the morph set itself. Since Set B roots do not impose any selectional restrictions on morph choice, the default form of each prefix is selected, illustrated in Table 11ii for *o-ki* tail.3.sg and in Table 11iii for 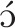-tu ear.6.plural.

Of some interest, however, and unexplained at this point, is why the morph set in Table 11iii includes the vowel [ε], since neither root Set A nor root Set C selects [ε], and since [ε] is not the default vowel for the morph set. To address this point, let us consider where { ε } appears with Set B items. Representative examples are given in Table 12i.

Inspection of these forms reveals a familiar restriction: not only must vowels agree for backness in roots but also, as these data show, in words as well. That is, two of the root restrictions seen above (^*^[back]…[front]_root_ and ^*^[front]…[back]_root_) hold more broadly than of the root alone. These two restrictions, stated in Table 12ii, hold of words, and so can drive the selection among morphs.

As seen in Table 12iii, the front/back requirement takes priority over default morph choice. Note that the front/back phonotactic serves to choose a particular morph, not to require morphs to change their form. Otherwise, the default [ɔ] is selected, Table 12iv. Where the morph set contains no morph satisfying the phonotactic condition, then the condition serves no deciding role. For example, since all class 9 morphs { i, e, ε } are front, it is impossible to satisfy the phonotactic when this prefix occurs with a back vowel root, e.g., [ì-sú] “fish” Tables 9i,iv, and other criteria determine which form to select.

#### 4.3.4. Excursus on identifying defaults

In this section, we consider how the default morph might be identified during acquisition. While completely arbitrary designation of a default morph may be necessary in at least some instances, there is more that can be said in general.

First, consider that the default morph must be in an elsewhere relation with selected morphs. For example, with the morph set { i, e, ε }, Set A roots select morph { i } and Set C roots select morph { ε }. In the absence of such specific selections, the default is therefore the only remaining morph, namely { e }. While the selected morphs must have specific properties to match selectional criteria, there is no such requirement of the default morph. We might therefore expect that in at least certain cases, *default morphs would not yield as straightforwardly to a unique characterization*. This is certainly true in the Esimbi case. The three default morphs { e }, { o }, and { ɔ } do not share any consistent features as unique identifiers of the set. They can be front, back, unrounded, rounded, advanced, retracted; even their mid-vowel height, while a necessary property in these prefix cases, is not a sufficient property (consider, for example, the set { o, ε, ɔ, a }).

Independent of such selectional issues, we might expect default morphs to exhibit certain properties. For example, all else being equal, we would expect that if morphs differ in their *frequency*: *the more frequent morph is the default morph*. While we consider this hypothesis reasonable, we do not have the data to assess it for Esimbi.

An additional property we hypothesize to hold of default morphs is *representability*, that is, *the default morph best represents the full set of morphs*. Consider three cases. If there is a single morph in a set, then obviously that morph is fully representative of the set. It is the “default” in that it will occur independent of specific requirements, but since there is only one form the notion of “default” is not interesting. If there are two morphs, then it is impossible to speak of one or the other better representing the set as each morph represents an identical (but opposite) divergence from the set's (putative) default. In such binary cases, we might refer to frequency to establish the default morph, but representability will be irrelevant. In cases with more than two morphs, however, we can assess overall properties of the morph set, and identify a particular morph as being representative of those properties.

We will consider the morph sets one by one, starting with the set { i, e, ε}. In this set, all vowels are front and unrounded. This clearly establishes that the prototypical version of this morph set should be front and unrounded. Differences in the morphs are restricted to differences in the features [high] and [ATR]. With respect to [high], two of the three vowels—the majority—are nonhigh while only one is high. This establishes that the prototypical value should be nonhigh. Similarly, two of the three morphs are advanced, establishing that the prototypical value should be advanced. Put together, a consideration of all features individually establishes that the prototypical morph for { i, e, ε} should be front, unrounded, high and advanced, that is, { e }.

A similar assessment of { u, o, ɔ } establishes that the prototypical morph in the morph set should be { o }. All vowels are back, all are rounded, two of the three are nonhigh, two of the three are advanced, hence back, rounded, nonhigh, advanced.

Turning to the four-vowel morph set { o, ε, ɔ, a }, the same assessment of representability establishes { ɔ } as the default. All vowels in the set are nonhigh. Three of the four vowels are nonlow. Three are back. Three are retracted. Interestingly, two of the vowels are rounded and two are unrounded. Hence a consideration of representability establishes that the prototypical vowel for this set should be nonhigh, nonlow, back, and retracted; rounding is not determined. A consideration of the prototypical properties uniquely identifies { ɔ } as the default (nonhigh, nonlow, back, retracted) in spite of the fact that rounding is indeterminate.

### 4.4. Summary: Opacity revisited

In this very brief discussion of Esimbi, we have shown that prefix forms in Esimbi have both idiosyncratic and systematic properties. The fact that there are three different prefix morph sets is idiosyncratic under this analysis, as is which prefix morph set is selected by a particular root. Each of these properties is characterized as part of the lexical representation for prefixes (morph set) and of roots (selection of prefix morph set). The choice of morphs from each set is systematic given the generalizations proposed for the language. The systematic properties are defined in terms of the features of the morphs, for specific selection within a class, for default selection, and for word-level phonotactic wellformedness.

The issue of surface opacity, raised in the discussion of Table 5b is a non-issue under this analysis. The problem derives from assuming that patterns such as these are entirely phonological. Assuming that a phonological difference in the roots is the source of the difference in prefix height requires that height distinctions be encoded in roots even though there is no surface evidence—in the roots—for the required distinction. “Markedness” constraints force uniformity of height features in roots; “faithfulness” constraints must reference features in roots which surface roots show no evidence of.

Emergent Grammar recognizes all types of generalizations that the learner might make. Among these are generalizations over sets of lexical items that are arbitrary based on their surface forms—such as a set of roots that selects for a high, advanced prefix. It is the recognition of such lexical generalizations co-existing with phonological generalizations that eliminates opacity as an issue in Esimbi prefix selection.

It is important to remember that Emergent Grammar principles led to this analysis of the interactions between phonology and morphology in Esimbi. At this point in our development of the model, learners identify morph sets, with selectional restrictions specific to morphs or morph sets, as well as purely phonological selectional criteria (such as the Esimbi prohibition against mixing back and front within a word). The generalizations proposed are all types of generalizations that might arise from making categories out of similar frequent items, coupled with a strong pressure to generalize and create a more abstract, symbolic representation.

## 5. Conclusion

We make three basic assumptions about human capabilities that are non-linguistic, but that are recruited to deal with language data:

a. Ability to create categories
b. Ability to attend to frequency
c. Ability to generalize and create a symbolic system

We presented three cases studies, each illuminating a way in which these capabilities are implemented in language. First, we considered the case of English /ɹ/, arguing for the spontaneous creation of categories and generalizations over those categories, even in the absence of external evidence. Second, we reviewed the implications of the frequency of specific patterns with respect to languages showing Bantu height harmony vs. languages without a height harmony pattern. Finally, we presented the case of Esimbi prefixes, showing the role of categorizing morphs into sets and generalizing over both morphological and phonological categories to select the appropriate prefix morph. This analysis also demonstrated the ability to characterize morphophonological interactions of considerable surface complexity, but without appealing to the power of complex innate linguistic capacities.

To conclude, we have argued that conceptually, there are good reasons to explore how far we can get without UG. As seen with our case studies, phonological analysis without appeal to UG has promising empirical coverage. In other words, assuming categorization, attention to frequencies, and a preference for generalization gets us a long way toward a phonological system with minimal appeal to innate linguistic-specific capabilities. In answer to the question raised by the topic of this issue, we have yet to discover a persuasive role for innate linguistic endowment in phonology of the type frequently assumed. At the same time, we find that Emergent exploration of the phonologies of different languages frequently reveals an interaction with lexical representations of morphs, suggesting there may be a largely Emergent component to language morphologies as well.

## Author contributions

The two authors shared equally in all aspects of this project.

## Funding

This research was supported in part by grant #410-2011-0230 to DP from the Social Sciences & Humanities Research Council of Canada.

### Conflict of interest statement

The authors declare that the research was conducted in the absence of any commercial or financial relationships that could be construed as a potential conflict of interest.

## Footnotes

^1^This section is based on a more complete study of Esimbi vowels (Archangeli and Pulleyblank, unpublished manuscript).

^2^The language is generally analyzed as having three level tones, a rising tone and a falling tone. Tone is marked as in our sources, though in some instances tones differ depending on the source and not all tones are marked orthographically. There are unresolved issues in the analysis of tone in Esimbi (Coleman et al., unpublished manuscript).

^3^These generalizations can be made even more general. For instance, *f* could be stated as “If a prefix has a high front vowel, its morph set includes all front vowels”; statements *f,h* can be generalized over: “If a prefix has a high vowel, its morph set includes all vowels with like rounding/backing,” and so on. However, further discussion of this type of generalization takes us afield from our main point here.

^4^While much of our proposal here is compatible with that of Donegan (2015), this is a difference: Donegan requires that the hearer “undo” phonological processes to access the lexical item; in our model, there is no single abstract underlying form and so no phonological processes to undo. In this way, our work is more similar to the Cognitive Grammar model in Nesset (2008).
